# Rosmarinic Acid Protects Against Acetaminophen-Induced Hepatotoxicity by Suppressing Ferroptosis and Oxidative Stress Through Nrf2/HO-1 Activation in Mice

**DOI:** 10.3390/md23070287

**Published:** 2025-07-14

**Authors:** Liqin Wu, Li Lv, Yifei Xiang, Dandan Yi, Qiuling Liang, Min Ji, Zhaoyou Deng, Lanqian Qin, Lingyi Ren, Zhengmin Liang, Jiakang He

**Affiliations:** Guangxi Key Laboratory of Animal Breeding, Disease Control and Prevention, College of Animal Science and Technology, Guangxi Zhuang Autonomous Region Engineering Research Center of Veterinary Biologics, Guangxi University, Nanning 530004, China; liqinwu16@126.com (L.W.); lvlilvli06@163.com (L.L.); 2318402002@st.gxu.edu.cn (Y.X.); 2118402010@st.gxu.edu.cn (D.Y.); qlliang1210@163.com (Q.L.); jimin1688@126.com (M.J.); pharmacologyvip@163.com (Z.D.); qlq1634130228@163.com (L.Q.); 2118393055@st.gxu.edu.cn (L.R.)

**Keywords:** rosmarinic acid, acetaminophen, liver injury, oxidative damage, ferroptosis

## Abstract

Liver injury caused by the irrational use of acetaminophen (APAP) represents a significant challenge in the field of public health. In clinical treatment, apart from N—acetylcysteine (NAC), the only approved antidote, there are extremely limited effective intervention measures for APAP-induced hepatotoxicity. Therefore, exploring novel liver-protecting drugs and elucidating their mechanisms of action is of great scientific significance and clinical value. Rosmarinic acid (RA), as a natural polyphenolic compound, has been proven to have significant antioxidant activity. Previous studies have shown that it has a protective effect against drug-induced liver injury. Nevertheless, the precise protective mechanism of RA in APAP-induced acute liver injury (AILI) has not been fully defined. This study was based on an AILI mouse model to systematically explore the liver-protecting effect of RA and its underlying molecular mechanisms. The research results showed that pretreatment with RA could notably mitigate liver pathological injury. It could decrease the activities of ALT and AST in the serum, suppress the liver inflammatory reaction, and reverse the decline in the levels of CAT, T-AOC, SOD, and GSH caused by APAP. Meanwhile, RA could enhance antioxidant defense capabilities by activating the Keap1/Nrf2/HO-1 signaling pathway, regulate the *xCT*/GPX4 axis to inhibit lipid peroxidation, and thus block the process of ferroptosis. In conclusion, this study confirmed that RA exerts a protective effect against AILI by regulating the Keap1/Nrf2/HO-1 axis to enhance antioxidant capacity and inhibit ferroptosis through the *xCT*/GPX4 pathway. Our research provides a theoretical basis for RA as a potential therapeutic agent for APAP-induced liver injury.

## 1. Introduction

Drug-induced liver injury (DILI) has become a crucial pathogenic factor for acute liver failure [1,2]. Prior research has indicated that DILI occurs at an annual rate of 14 to 19 cases per 100,000 individuals, and approximately 20% of pediatric acute liver failure cases are associated with it [3]. Excessive intake of acetaminophen (APAP), a widely used antipyretic and analgesic drug, is the main cause of DILI [4]. Currently, only N–acetylcysteine (NAC) has been approved clinically as an antidote. It exerts its detoxification effect by promoting the regeneration of glutathione (GSH) in the liver. However, NAC also has various side effects. It is only effective when taken within 8–10 h after an overdose of APAP [5]. Therefore, the development of a new, safe, and highly effective antidote for APAP-induced acute liver injury (AILI), i.e., acute liver injury caused by acetaminophen, is extremely urgent.

An overdose of APAP may lead to substantial oxidative damage in the liver [6]. Roughly 10% of APAP undergoes metabolism via the cytochrome P450 enzyme system (CYP), generating the toxic metabolite N-acetyl-p-benzoquinone imine (NAPQI). The Cytochrome P450 2E1 (*CYP2E1*) subtype serves as the primary catalytic enzyme in this process. When the GSH reserve is depleted, NAPQI builds up in the liver tissue, covalently binds to mitochondrial proteins, and leads to the excessive production of reactive oxygen species (ROS) [7]. This oxidative stress state deranges the equilibrium of pro-inflammatory and anti-inflammatory reactions by activating the innate immune response, ultimately inflicting damage on liver tissue. As a core regulatory factor of the oxidative stress defense system [8,9], the nuclear factor erythroid 2–related factor 2 (Nrf2) bolsters the cells’ detoxification capacities by upregulating the expression levels of antioxidant enzyme systems, such as heme oxygenase-1 (HO-1) and NAD(P)H: quinone oxidoreductase 1 (NQO1). Recent studies suggest that the Kelch-like ECH-associated protein 1 (Keap1)-Nrf2 signaling pathway plays a critical role in regulating ferroptosis [8,9]. The downstream targets of this pathway, solute carrier family 7 member 11 (*xCT*) and glutathione peroxidase 4 (GPX4), inhibit lipid peroxidation by maintaining glutathione levels, thus blocking the process of ferroptosis. Notably, ferroptosis has been verified to be implicated in the death of primary hepatocytes triggered by APAP, and antioxidant intervention can effectively alleviate AILI [10]. This implies that targeting the antioxidant stress system could represent a promising therapeutic strategy for managing AILI.

Rosmarinic acid (RA) is a natural phenolic compound derived from various marine plants, such as the *Gracilariopsis persica* [11] and *Halodule pinifolia* (Seagrass) [12], as well as *Phyllospadix* (Zosteraceae) [13]; it possesses multiple pharmacological activities, such as antioxidant, hepatoprotective, and anti-inflammatory effects [14,15]. Prior investigations from our group have demonstrated that RA effectively mitigates ovalbumin-induced airway inflammation and cisplatin-associated hepatorenal injury through modulation of the NF-κB and Nrf2 signaling axes, alongside regulation of antioxidant enzyme activities [14,16,17]. Notably, oxidative stress and lipid peroxide accumulation in the liver induced by APAP overdose are closely interconnected pathological factors. Prior research has demonstrated that RA effectively attenuates AILI, suggesting its potential role in addressing these associated mechanisms [14,18], but its specific mechanism remains unclear. Recent research has demonstrated that the Nrf2/HO-1 signaling pathway is pivotal in regulating ferroptosis [19,20], and whether RA can intervene in AILI by regulating this pathway still needs in-depth exploration.

## 2. Results

### 2.1. APAP Caused Liver Injury

**Figure 1:**
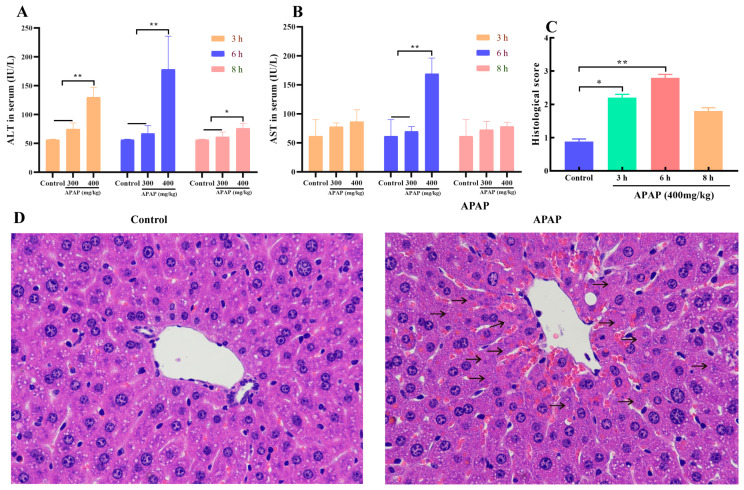
The AILI model in mice induced by APAP (*n* = 10). (**A**,**B**): Serum ALT and AST activities at 3, 6, and 8 h post-APAP administration. (**C**): Histological injury scores of liver tissue. (**D**): Representative HE-stained liver tissue section images (×400). Arrows represent the typical necrotic hepatic cells. Mice were administrated with APAP (400 mg/kg) for 6 h. * and ** indicate significant difference (*p* < 0.05) and extremely significant difference (*p* < 0.01) respectively, both of which are statistically significant.

Alanine aminotransferase (ALT) and aspartate aminotransferase (AST) activities serve as sensitive biomarkers of hepatocyte injury. In comparison to the control group, APAP administration significantly elevated serum ALT and AST levels. Additionally, enzyme activities were significantly higher in the 400 mg/kg vs. 300 mg/kg APAP group (*p* < 0.01). A time-dependent analysis showed that the peak of enzyme activities occurred 6 h after administration (Figure 1A,B). A histological analysis indicated that, in contrast to the control group, the group treated with APAP exhibited obvious hepatocyte necrosis (as indicated by the arrow) around the central vein (Figure 1C,D) (*p* < 0.05). The above data confirmed that treating mice with 400 mg/kg of APAP for 6 h could successfully induce acute liver injury in mice.

### 2.2. RA Mitigated APAP-Induced Hepatic Injury

**Figure 2:**
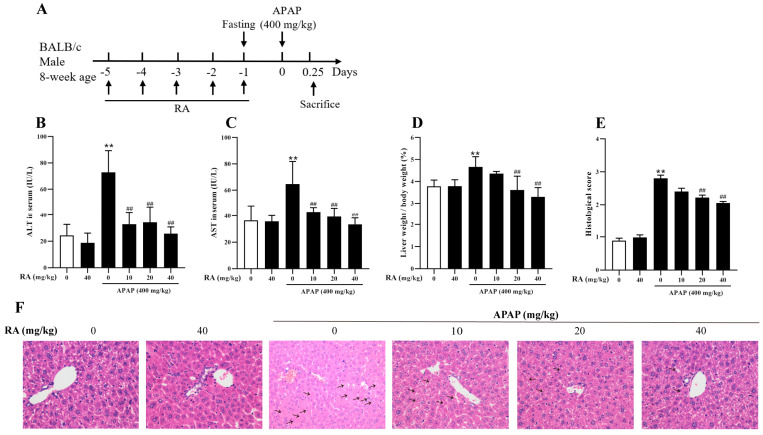
RA alleviates APAP-induced hepatic injury in mice (*n* = 6). (**A**): Experimental protocol schematic. (**B**,**C**): The concentrations of ALT and AST present in the serum. (**D**): Liver Index, Liver Index (%) = (Liver Weight [g]/Body Weight [g]) × 100%. (**E**): Histopathological injury scores of liver tissue. (**F**): Representative HE-stained liver tissue section images (×200). Arrows represent the typical necrotic hepatic cells. ** indicates an extremely significant difference compared with the control group (*p* < 0.01); ## indicates an extremely significant difference compared with the model group (*p* < 0.01). All these differences are statistically significant.

To investigate the protective effects of RA against APAP-induced hepatotoxicity, the mice underwent RA pretreatment prior to APAP challenge (Figure 2A). The serum biochemical assays showed that APAP notably increased the levels of ALT and AST, whereas RA pretreatment attenuated these enzyme activities (Figure 2B,C) (*p* < 0.01). Concurrently, APAP caused a significant increase in the liver index, which was effectively inhibited by RA pretreatment at 20–40 mg/kg (Figure 2D) (*p* < 0.01), indicating RA’s role in reducing liver edema. Histopathological examination via HE staining showed that APAP induced severe hepatocyte necrosis, while 40 mg/kg of RA pretreatment markedly mitigated tissue damage, obviously decreasing the number of necrotic hepatic cells (as indicated by the arrow) (Figure 2E,F). Additionally, compared to the control group, administration of RA alone at a dose of 40 mg/kg exerted no significant effects on the serum biochemical parameters, liver index, and hepatic histopathological manifestations in the mice. Collectively, these findings demonstrate that RA exerts protective efficacy against APAP-induced liver injury.

Regarding the explanation of the grouping (which is consistent throughout the text), taking Figure 2B as an example, from left to right, the groups are represented as the control group (0), the RA-alone group (40), and the RA pretreatment (0, 10, 20, 40) + APAP challenge groups.

### 2.3. RA Regulated the APAP-Induced Inflammatory Response

**Figure 3:**
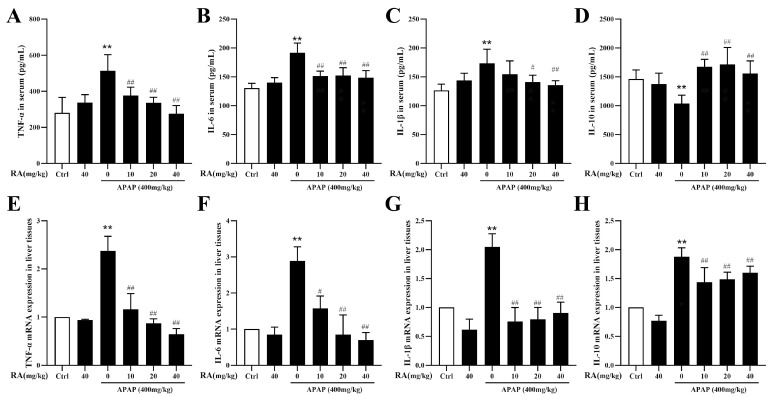
RA alleviates APAP-induced hepatic inflammation (*n* = 6). (**A**–**D**): Serum concentrations of TNF-α, IL-1β, IL-6, and IL-10 were determined by ELISA. (**E**–**H**): Hepatic mRNA levels of TNF-α, IL-1β, IL-6, and IL-10 were assessed via RT-qPCR. ** indicates a highly significant difference compared with the control group (*p* < 0.01), # and ## indicate a significant (*p* < 0.05) or highly significant (*p* < 0.01) difference compared with the model group, respectively, all of which are statistically significant.

The serum and liver tissue levels of pro- and anti-inflammatory cytokines were assessed using ELISA and RT-qPCR. The findings revealed that, compared to the control group, administration of 40 mg/kg of RA alone exerted no significant effects on the levels of inflammatory cytokines in the mice. By contrast, the APAP-treated group showed notably increased serum levels of tumor necrosis factor-α (TNF-α), interleukin-1β (IL-1β), and interleukin-6 (IL-6), along with significantly decreased interleukin-10 (IL-10) levels (*p* < 0.01). Conversely, RA pretreatment suppressed pro-inflammatory cytokines’ expression (TNF-α, IL-1β, IL-6) and enhanced anti-inflammatory IL-10 levels (Figure 3A–D) (*p* < 0.05). A liver tissue gene expression analysis revealed that RA significantly inhibited mRNA transcription of TNF-α, IL-1β, and IL-6 while promoting IL-10 gene expression (Figure 3E–H) (*p* < 0.05). Collectively, these findings demonstrate that RA effectively mitigates APAP-induced hepatic inflammation by regulating cytokine balance.

### 2.4. RA Alleviated APAP-Triggered Oxidative Damage

**Figure 4:**
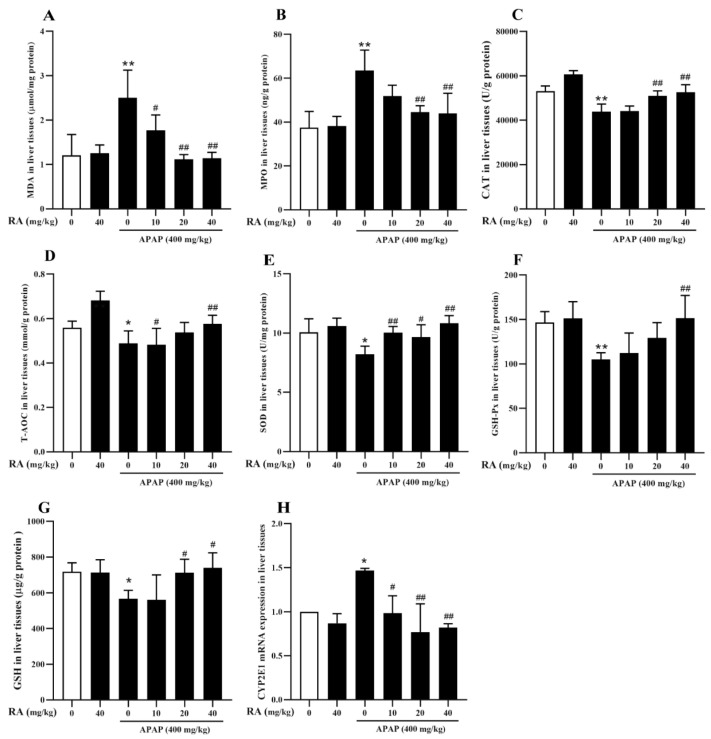
RA exerts protective roles against APAP-mediated hepatic oxidative stress (*n* = 6). (**A**–**G**): Measurements of MDA, MPO, CAT, T-AOC, SOD, GSH-Px, and GSH in hepatic tissues. (**H**): Analysis of *CYP2E1* mRNA expression in hepatic tissues. * and ** indicate a significant difference (*p* < 0.05) or an extremely significant difference (*p* < 0.01) compared with the control group, respectively; # and ## indicate a significant difference (*p* < 0.05) or an extremely significant difference (*p* < 0.01) compared with the model group, respectively. All these differences are statistically significant.

To evaluate the protective roles of RA in counteracting APAP-mediated oxidative liver injury, antioxidant enzyme activities and oxidative stress markers in liver tissues were measured. Compared with the control group, administration of 40 mg/kg of RA alone had no significant effects on the antioxidant-related indices in the mice. In contrast, APAP administration significantly elevated malondialdehyde (MDA) and myeloperoxidase (MPO) levels while reducing catalase (CAT), total antioxidant capacity (T-AOC), Superoxide dismutase (SOD), glutathione peroxidase (GSH-Px), and GSH levels (*p* < 0.05). Conversely, 40 mg/kg of RA pretreatment significantly reversed these alterations (Figure 4A–G) (*p* < 0.01). *CYP2E1*, a critical enzyme in the Cytochrome P450 family, catalyzes the generation of toxic metabolites from APAP. This study found that APAP could significantly upregulate the mRNA transcription of the *CYP2E1* gene, while pretreatment with RA could significantly inhibit the increase in *CYP2E1* mRNA expression induced by APAP (Figure 4H) (*p* < 0.01). In conclusion, these results indicate that RA mitigates hepatic oxidative stress by modulating antioxidant parameters and inhibiting *CYP2E1* gene expression during APAP intoxication.

### 2.5. RA Ameliorates APAP-Mediated Oxidative Damage by Regulating the Keap1/Nrf2/HO-1 Pathway

**Figure 5:**
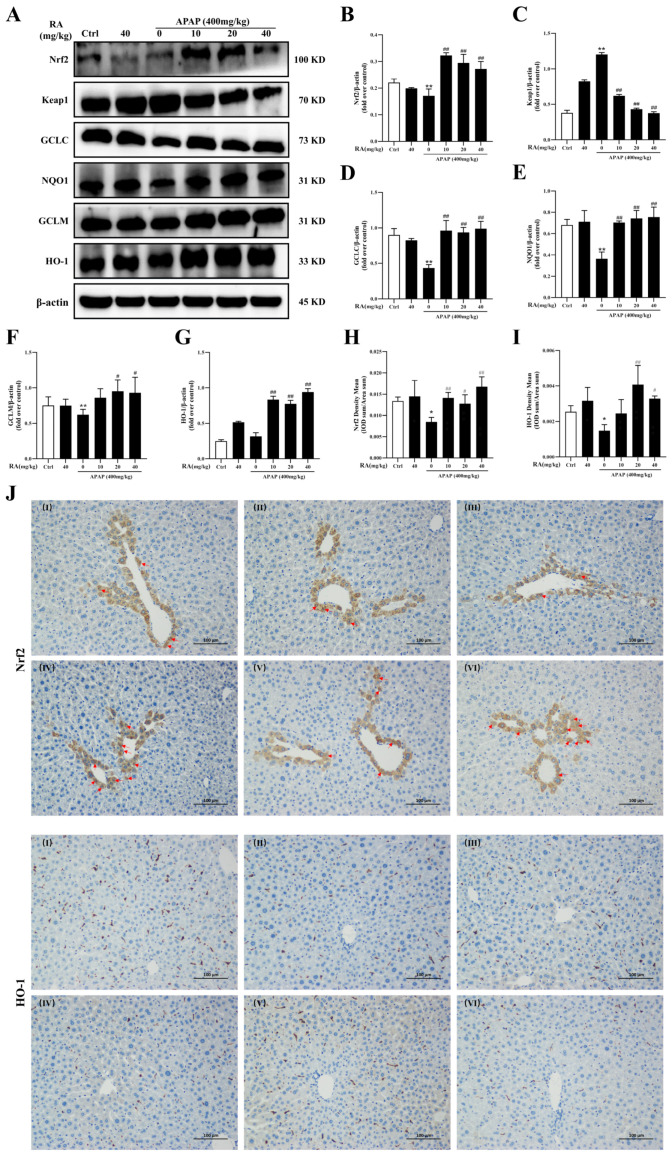
RA modulates the Keap1/Nrf2/HO-1 pathway to mitigate APAP-induced hepatic injury (*n* = 6). (**A**): Representative Western blotting bands of Nrf2, Keap1, GCLC, NQO1, GCLM, HO-1, and the internal reference β-actin in liver tissues. (**B**–**G**): Relative protein expression levels standardized using β-actin as the internal reference. (**H**,**I**): Quantitative immunohistochemical analysis of Nrf2 and HO-1. (**J**): Representative immunohistochemical images of Nrf2 and HO-1 in liver sections (×200), and the proportion of the positive area was analyzed by ImageJ software (1.5.4p). (**I**) Control group, (**II**) RA (40 mg/kg) group, (**III**) APAP (400 mg/kg) group, (**IV**) RA (10 mg/kg) + APAP group, (**V**) RA (20 mg/kg) + APAP group, and (**VI**) RA (40 mg/kg) + APAP group. Arrows represent the typical hepatic cells expressing Nrf2 in the nucleus. * and ** indicate a significant difference (*p* < 0.05) or an extremely significant difference (*p* < 0.01) compared with the control group, respectively; # and ## indicate a significant difference (*p* < 0.05) or an extremely significant difference (*p* < 0.01) compared with the model group, respectively. All these differences are statistically significant.

To explore RA’s regulation of the Keap1/Nrf2/HO-1 pathway during APAP-mediated hepatic injury, the expression levels of related proteins were analyzed. Keap1 functions as a negative regulator of Nrf2, with downstream targets including HO-1, glutamate-cysteine ligase catalytic subunit (GCLC), glutamate-cysteine ligase modifier subunit (GCLM), and NQO1. A Western blot analysis revealed that APAP treatment significantly reduced hepatic Nrf2 protein expression and increased Keap1 levels compared to the control group, effects reversed by RA pretreatment (Figure 5A–C) (*p* < 0.01). Additionally, RA enhanced expression of the Nrf2 downstream genes GCLC, NQO1, GCLM, and HO-1 (Figure 5A,D–G) (*p* < 0.05). Immunohistochemical staining showed decreased positive staining for Nrf2 and HO-1 in the APAP group (Figure 5H–J) (*p* < 0.05), which was notably elevated by RA pretreatment, and the typical hepatic cells expressing Nrf2 in the nucleus were obviously increased. Collectively, these results indicate that RA alleviates APAP-mediated hepatic oxidative damage by activating the Nrf2 signaling cascade and its downstream effectors.

### 2.6. RA Inhibits APAP-Induced Hepatocyte Apoptosis

**Figure 6:**
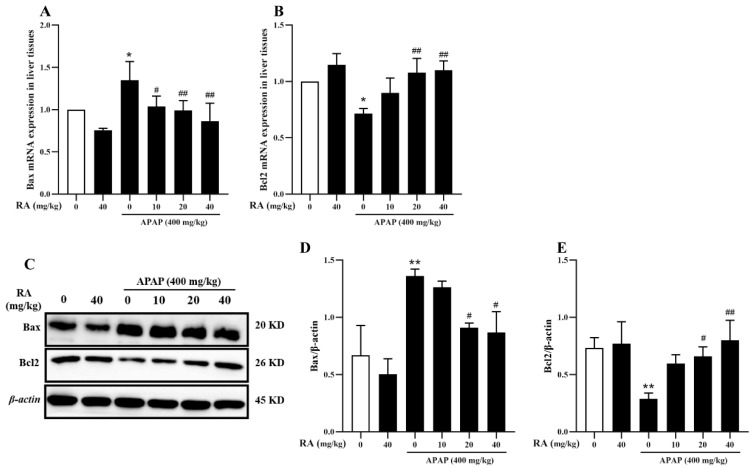
RA mitigates APAP-induced hepatic tissue apoptosis (*n* = 6). (**A**,**B**): RT-qPCR examination of *Bax* and *Bcl2* mRNA in hepatic tissues. (**C**): Representative Western blot images of *Bax*, *Bcl2*, and β-actin in hepatic tissues. (**D**,**E**): Relative protein expression levels of *Bax* and *Bcl2* normalized to β-actin in hepatic tissues. * and ** indicate a significant difference (*p* < 0.05) or an extremely significant difference (*p* < 0.01) compared with the control group, respectively; # and ## indicate a significant difference (*p* < 0.05) or an extremely significant difference (*p* < 0.01) compared with the model group, respectively. All these differences are statistically significant.

Excessive APAP intake causes oxidative stress alongside mitochondrial damage, resulting in hepatocyte death. Among them, apoptosis and necrosis are the key cell death pathways in DILI [21,22]. The expressions of the BCL-2 associated X protein (*Bax*), and B-cell lymphoma-2 (*Bcl2*) were analyzed. Specifically, APAP upregulated *Bax* mRNA and protein levels while reducing *Bcl2* expression compared to the control group; these alterations were significantly reversed by RA pretreatment (Figure 6) (*p* < 0.05). These findings suggest that RA modulates *Bax*/*Bcl2* balance to inhibit APAP-induced hepatocyte apoptosis.

### 2.7. RA Alleviates APAP-Induced Ferroptosis

**Figure 7:**
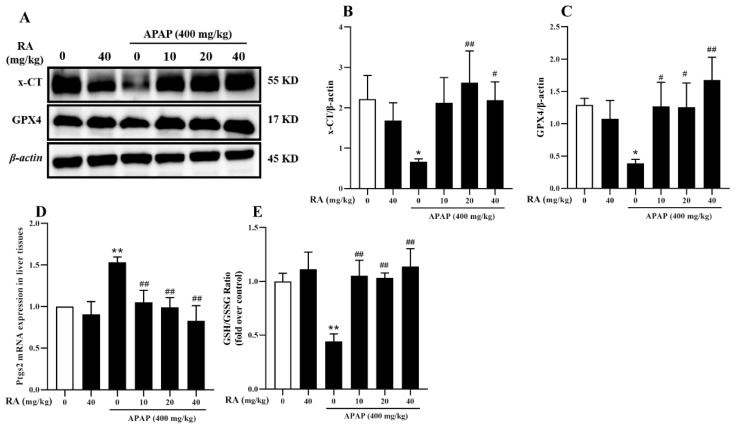
RA alleviates APAP-induced ferroptosis in liver tissues (*n* = 6). (**A**): Typical Western blot bands for *xCT*, GPX4, and the loading control β-actin in hepatic tissues. (**B**,**C**): *xCT* and GPX4 protein levels in hepatic tissues were normalized against β-actin. (**D**): RT-qPCR analysis of *Ptgs2* mRNA in hepatic tissues. (**E**): The ratio of GSH to GSSG in liver tissues. * and ** indicate a significant difference (*p* < 0.05) or an extremely significant difference (*p* < 0.01) compared with the control group, respectively; # and ## indicate a significant difference (*p* < 0.05) or an extremely significant difference (*p* < 0.01) compared with the model group, respectively. All these differences are statistically significant.

Ferroptosis is an iron-related cell death pattern, which is characterized by lipid peroxidation and the shrinkage of mitochondria [23], and is implicated in APAP-induced hepatocyte injury, with GPX4 and *xCT* inhibition identified as key triggers [24,25]. To investigate RA’s role in APAP-induced ferroptosis, Western blotting was used to analyze *xCT* and GPX4 protein levels, alongside *Ptgs2* mRNA expression and the GSH/glutathione disulfide (GSSG) ratio. The results showed APAP significantly downregulated *xCT* and GPX4 compared to the control group, effects which were reversed by RA pretreatment (Figure 7A–C) (*p* < 0.05), suggesting RA promotes GPX4 expression via *xCT* upregulation. Additionally, RA reduced APAP-induced *Ptgs2* mRNA levels and increased the GSH/GSSG ratio (Figure 7D,E) (*p* < 0.01). Collectively, these findings indicate RA inhibits APAP-mediated ferroptosis through regulation of the *xCT*/GPX4 axis.

## 3. Discussion

As one of the most commonly used analgesic and antipyretic drugs worldwide, APAP is available over-the-counter in both single-ingredient formulations and multi-component preparations. It often serves as the first-line medication for patients when non-steroidal anti-inflammatory drugs (NSAIDs) are contraindicated due to other medical conditions. However, excessive use or long-term cumulative exposure may lead to severe complications such as DILI [1,26]. DILI is a primary cause of acute liver failure, with its pathological progression involving hepatocyte necrosis or programmed cell death [1,2]. Recent studies have identified an association between ferroptosis—a subtype of regulated cell death—and AILI [24,25], although the specific intervention mechanisms remain to be further elucidated.

Natural products, leveraging their multi-target regulatory profiles and favorable safety profiles [27], exhibit significant promise in managing APAP-induced liver toxicity. As a representative phenolic compound, RA has been shown to exert protective effects in DILI mouse models in prior research [14,18], yet its molecular mechanisms remain incompletely defined in vivo. Using an APAP-induced hepatotoxicity murine model, this study demonstrated that RA effectively mitigated hepatic inflammatory responses and oxidative damage by lowering serum ALT/AST activities, suppressing pro-inflammatory cytokine expression, and enhancing antioxidant enzyme activity. While HepG2 cell models have implicated the Nrf2 and NEK7-NLRP3 pathways in APAP-induced liver toxicity in previous studies [18], this in vivo investigation further confirmed that RA exerts protective effects by regulating multiple pathways, including Nrf2, *Bax*-*Bcl2*, *xCT*, and GPX4, thereby expanding the understanding of the multi-pathway mechanisms underlying RA’s hepatic protective effects.

As a pivotal transcription factor orchestrating oxidative stress responses, Nrf2 maintains cellular redox homeostasis through a conserved molecular mechanism by upregulating the synthesis of antioxidant proteins, such as NQO1 [28]. Under physiological conditions, Nrf2 is sequestered in the cytoplasm by Keap1 in an inactive complex. Upon exposure to oxidative stressors, such as ROS, Nrf2 dissociates from Keap1, translocates into the nucleus, and initiates the transcription of target genes harboring antioxidant response elements in their promoters [18,29]. This study reveals that APAP potently suppresses the Nrf2 pathway via the upregulation of Keap1 protein expression, whereas RA pretreatment markedly reverses this inhibition. These findings collectively indicate that RA alleviates APAP-mediated liver injury by activating the Keap1/Nrf2/HO-1 signaling axis, underscoring the critical role of this pathway in RA’s hepatoprotective effects.

Ferroptosis, a distinct subtype of regulated cell death distinguishable from apoptosis and necrosis [30], is characterized by an iron-dependent accumulation of lipid peroxidation products, with *xCT* and GPX4 serving as key molecular regulators [31]. Emerging evidence has established ferroptosis as a central driver in the pathogenesis of liver injury [32,33]. Building on this mechanistic framework, the present study hypothesized that RA might attenuate ferroptosis through the activation of the Nrf2/HO-1 pathway. This study demonstrated that RA effectively suppressed APAP-triggered ferroptosis by upregulating *xCT* and GPX4 protein expression, representing a novel insight into the hepatoprotective mechanisms of RA. Concurrently, RA modulated the balance of pro-apoptotic *Bax* and anti-apoptotic Bcl-2 proteins, further highlighting its multifunctional role in regulating programmed cell death pathways beyond ferroptosis. Although, a previous study showed that 20–80 mg/kg of RA protected APAP-induced hepatotoxicity in vivo, and found that RA may achieve a hepatoprotective effect by inhibiting apoptosis and activating Nrf2 to in vitro [18]. In the current study, we found that 10 mg mg/kg of RA could also protect against APAP-induced hepatotoxicity, and confirmed that RA activated the Nrf2/ HO-1pathway and suppressed apoptosis and ferroptosis in vivo. However, the relationship between the RA-regulated Nrf2 signaling pathway and apoptosis as well as ferroptosis will be further clarified, and further investigations focusing safety and the effectiveness of clinical application will be necessary.

In conclusion, RA has shown a good liver-protecting effect in the APAP-induced AILI model, effectively relieving the liver inflammatory response and significantly enhancing the body’s ability to resist oxidative damage. The core protective effect of RA operates through regulation of the Nrf2/HO-1/ferroptosis signaling pathway. These findings highlight RA as a promising, safe, and effective hepatoprotective agent, providing a robust foundation for future clinical translation research.

## 4. Materials and Methods

### 4.1. Reagents and Chemicals

Antibodies against Keap1, Nrf2, HO-1, NQO1, GCLC, GCLM, *Bax*, *Bcl2*, *xCT*, GPX4, and β—actin were supplied by Cell Signaling Technology (Boston, MA, USA). Additionally, the assay kits for ALT, AST, MDA, MPO, GSH, CAT, T-AOC, GSH-Px, and SOD came from the Nanjing Jiancheng Bioengineering Institute (Nanjing, China). The detection kits for GSH and GSSG were obtained from Beyotime Biotechnology (Nantong, China).

### 4.2. Animal Experiment Setup

Six- to eight-week-old male BALB/c mice (18–20 g) were obtained from the Hunan SJA Laboratory Animal Co., Ltd. (Changsha, China; SCXK2019-0004). During the experiment, the mice had ad libitum access to water and chow; the environmental temperature was maintained at approximately 25 ± 1 °C, and a 12 h light-dark cycle was applied.

To establish an APAP-induced liver injury model in BALB/c mice, the methodology from previous studies was referenced [34,35]; the mice were randomly divided into 3 groups (10 mice per group), including a blank control group, and APAP (300 mg/kg and 400 mg/kg) groups. The mice in each group were intraperitoneally injected with 300 mg/kg or 400 mg/kg of APAP (Sigma-Aldrich, St. Louis, MO, USA), while the control group received an equal volume of physiological saline. Serum and liver tissues were collected at 3, 6, and 8 h post-administration. Serum ALT and AST levels were measured by biochemical analysis, and a histological comprehensive evaluation of liver injury was performed using hematoxylin-eosin (HE) staining to establish an AILI mouse model.

Subsequently, an intraperitoneal injection of 400 mg/kg of APAP for 6 h was identified as the optimal model dose. To evaluate the protective effect of RA (HPLC ≥ 98%; Chengdu Pufei De Biotech Co., Ltd., Chengdu, China) against APAP-induced liver injury, 36 mice were randomly divided into 6 groups (i.e., a control group, an RA-alone group, and different doses of RA pretreatment + APAP challenge groups), with 6 mice per group. The mice were intraperitoneally administered with normal saline or RA (10, 20, or 40 mg/kg) daily for 5 consecutive days, followed by a single injection of 400 mg/kg of APAP. At 6 h after APAP administration, the mice were humanely euthanized, and samples were collected for analysis. Additionally, the safety of 40 mg/kg of RA in mice was evaluated by separate administration. Throughout the experiment, the control mice received an equal volume of normal saline.

### 4.3. Histopathological and Immunohistochemical Examination

For histopathological analysis, liver tissues were harvested at 3, 6, and 8 h post-APAP treatment. The excised liver tissues were rinsed with normal saline and fixed in a 10% formalin fixative for 48 h. Subsequently, they were dehydrated in gradient ethanol, embedded in paraffin, and cut into 5 μm thick sections. These sections were baked at 65 °C for 4.5 h. Following dewaxing and dehydration, the tissue sections were stained with HE and then mounted. Finally, a pathological analysis was carried out using an optical microscope. The degree of liver injury was scored on a scale of 0–3 based on a previous study [36].

Liver tissues were harvested 6 h post-APAP treatment to detect the expression of Nrf2 and HO-1 via immunohistochemistry. Paraffin sections were dewaxed by baking at 60 °C for 20 min and then hydrated sequentially with xylene and gradient ethanol (from 100% to 70%). Antigen retrieval was carried out using proteinase K (20 μg/mL). Endogenous enzyme activity was inhibited by using 3% hydrogen peroxide (H_2_O_2_). The sections were initially incubated with 3% bovine serum albumin (BSA) for 30 min to prevent non-specific binding. Subsequently, they were incubated at 4 °C overnight with primary antibodies targeting Nrf2 or HO-1, which were diluted 1:1000 in 3% BSA. Following a washing step, the sections were exposed to a horseradish peroxidase (HRP)-labeled secondary antibody for 30 min, developed with DAB for 10 min, and counterstained with hematoxylin. Neutral resin was used for slide mounting. After image acquisition under a microscope, the Image-Pro Plus 6.0 analysis software was employed to analyze the expression levels of target proteins in the tissues (Media Cybernetics, Inc., Bethesda, MD, USA).

### 4.4. Measurement of Biomarkers of Oxidative Stress

Liver and blood samples were harvested 6 h following APAP administration to assess biochemical parameters. Serum ALT and AST activities were quantified by ELISA. After homogenizing the liver tissues, the levels of MPO, CAT, T-AOC, GSH-Px, GSH, MDA, SOD, and GSH/GSSG were measured using commercial kits following the manufacturers’ instructions.

### 4.5. ELISA

Blood was collected 6 h after APAP treatment and then centrifuged to collect the serum. The serum levels of TNF-α, IL-6, IL-10, and IL-1β were assayed via ELISA kits from Neobioscience, Beijing, China, as per the manufacturer’s protocols.

### 4.6. RNA Isolation and RT-qPCR Analysis

Liver tissue total RNA was extracted by RNAiso Plus (Takara Bio Inc., Otsu, Japan), and 1 μg of RNA was used as the template to synthesize cDNA with the PrimeScript RT-PCR Kit (Takara, Dalian, China). RNA purity was assessed via a micro ultraviolet spectrophotometer, with an A260/A280 ratio of 1.9–2.1 confirming suitable quality. The primer sequences of the corresponding genes are presented in Table 1. RT-qPCR was performed using the Bio-Rad SYBR^®^ Premix Ex Taq™ II system (Richmond, CA, USA). The 2^−ΔΔCt^ method was utilized for relative quantification.

### 4.7. Western Blot Analysis

Total liver tissue proteins were isolated using a commercially available kit (Shanghai Biyuntian Biological Co., Ltd., Shanghai, China) according to the manufacturer’s instructions. Protein concentration was determined by the BCA method, and 20 μg of each sample was prepared for analysis. Target proteins were separated by 10% SDS-PAGE and electrotransferred to PVDF membranes. After transfer, the PVDF membranes were blocked with 5% skim milk on a shaker at room temperature for 2 h, washed three times with TBST (5 min each), and then cut into strips based on target protein molecular weights. Membranes were incubated with primary antibodies (Wuhan Servicebio Technology Co., Ltd., Wuhan, China) overnight at 4 °C, washed three times with TBST, and probed with secondary antibodies (Cell Signaling Technology, Inc., Boston, MA, USA) at 37 °C for 1 h. Following three additional TBST washes (5 min each), proteins were visualized using an ECL chemiluminescent solution (Cytiva) and exposed for imaging. Densitometric quantification of protein expression was performed by grayscale integration analysis using ImageJ software (National Institutes of Health, Bethesda, MD, USA), with β-actin serving as the loading control.

### 4.8. Data Analysis

Each experiment was replicated in triplicate and the data derived therefrom were collected and calculated with Excel. All data were tested for normal distribution with GraphPad Prism 10.1.2 (GraphPad Software Inc., La Jolla, CA, USA), unless clearly stated otherwise. For normally distributed data, one-way ANOVA and Tukey’s multiple comparison test were used to analyze and compare the significance of experimental findings, followed by statistical processing and graph plotting. Statistical significance was defined as a *p*-value < 0.05, where * indicates *p* < 0.05; ** indicates *p* < 0.01 vs. control group; and ^#^ indicates *p* < 0.05, ^##^ indicates *p* < 0.01 vs. APAP group.

## Figures and Tables

**Table 1:** RT-qPCR primer sets.

Targets	Forward	Reverse
IL-1β	AAAAAAGCCTCGTGCTGTCG	GTCGTTGCTTGGTTCTCCTTG
IL-6	CTAGTGCGTTATGCCTAAGC	ATAGTGTCCCAACATTCATATTGTC
IL-10	CAAGGCCATGAATGAATTTGACATC	TTCGGAGAGAGGTACAAACGAGGTT
TNF-α	CGCTGAGGTCAATCTGC	GGCTGGGTAGAGAATGGA
*CYP2E1*	GACGTGCGGAGGTTTTC	GCTGGCCTTTGGTCTTTT
*Ptgs2*	AAATGCTGGTGTGGAAGGT	TTGTTGCTCTAGGCTTTGCT
*Bax*	CTGGATCCAAGACCAGGGTG	GTGAGGACTCCAGCCACAAA
Bcl-2	AAACCCTCCATCCTGTCC	TCCTAAACCCTGCTTCCC
β-actin	ATCACTATTGGCAACGAGCG	TCAGCAATGCCTGGGTACAT

## Data Availability

The data that support the findings of this study are available from the corresponding author upon reasonable request.

## Abbreviations

The following abbreviations are used in this manuscript:

AILI	Acute liver injury caused by acetaminophen
APAP	Acetaminophen
DILI	Drug-induced liver injury
RA	Rosmarinic acid
